# A Novel Method of a High Pressure Processing Pre-Treatment on the Juice Yield and Quality of Persimmon

**DOI:** 10.3390/foods10123069

**Published:** 2021-12-10

**Authors:** Jiayue Xu, Yilun Wang, Xinyue Zhang, Zhen Zhao, Yao Yang, Xin Yang, Yongtao Wang, Xiaojun Liao, Liang Zhao

**Affiliations:** 1 National Engineering Research Center for Fruit and Vegetable Processing, Key Laboratory of Fruit and Vegetable Processing, Key Laboratory of Food Non-Thermal Processing, Ministry of Agricultural and Rural Affairs, College of Food Science & Nutritional Engineering, China Agricultural University, Beijing 100083, China; xujiayue@cau.edu.cn (J.X.); wangyilun@yili.com (Y.W.); zhangxinyuezy@163.com (X.Z.); yousafzai823@163.com (Z.Z.); yy1221yhy@163.com (Y.Y.); 2018306100512@cau.edu.cn (X.Y.); wangyongtao102@163.com (Y.W.); liaoxjun@cau.edu.cn (X.L.); 2 Xinghua Industrial Research Centre for Food Science and Human Health, China Agricultural University, Xinghua 225700, China

**Keywords:** persimmon, juice yield, high pressure processing, pectin methylesterase, enzyme activation, quality enhanced

## Abstract

This study investigates the effects of a high pressure processing pre-treatment (pre-HPP) on the juice yield of persimmon (*Diospyros kaki* L.) pulp and the pre-HPP plus HPP or thermal processing (TP) on microorganism inactivation and quality changes of the persimmon juice. The “Gongcheng” persimmon was selected with the highest juice yield (48.9%), and the pre-HPP set at 300 MPa/8 min increased the juice yield by 60% through an increasing pectin methylesterase (PME) activity of 25.03% and by maintaining polygalacturonase (PG) activity. For different processing modes, namely, pre-HPP plus HPP at 550 Mpa/5 min and pre-HPP plus TP treatment at 95 °C/5 min, both of the guaranteed microorganisms in the juice were below 2.0 lg CFU/mL; however, the persimmon juice treated by the pre-HPP plus HPP had higher contents of total phenol and ascorbic acid which were 16.07 mg GAE/100 g and 17.92 mg/100 mL, respectively, a lower content of soluble tannin which was 55.64 μg/mL, better clarity which was 18.6% and less color change where the Δ*E* was only 2.68.

## 1. Introduction

Persimmon (*Diospyros kaki* L.) is one of the most popular fruits in China. In 2017, China’s persimmon yield was 3.25 million tons, accounting for 76% of the world’s yield [1]. Although the harvest time of persimmon is short and it is a difficult fruit to transport, only one-tenth of those were able to be processed into dried persimmons and other products. Since the large amounts of tannin, pectin and protein in persimmon would affect the quality and the juice yield, persimmon juice products have rarely existed in the market.

A large amount of pectin could cause a low juice yield which is one of the main problems of persimmon juice processing. A traditional way to solve this problem is by adding an exogenous pectinase, such as PME and PG, which could increase the fruit juice yield, clarify the juice and increase membrane flux during ultrafiltration. In previous research, pectinase was added in the processing of juice with enzymatic hydrolysis at the appropriate time and temperature [2]; however, the main disadvantages of adding exogenous pectinase are its hidden safety perils and a complexity in formulating the optimum enzyme mixture, that also has a bad effect on the taste of the juice [3].

High pressure processing (HPP) is widely used in the food industry as a non-thermal technology, especially in fruits or vegetable products. Studies have indicated that compared to traditional thermal processing (TP), HPP efficiently enhances the microbial inactivation and enzyme denaturation with less damage on the low molecular weight compounds, like vitamins and flavoring agents [4]. However, some enzymes in food are pressure resistant and are even activated after HPP [5]. Several mechanisms were proposed for the pressure-induced activation of enzyme-catalyzed reactions: (1) changes in the structure of the enzyme; (2) accelerating the reaction rate of the enzyme; and (3) changes in the substrate or solvent’s physical properties (such as pH, density, viscosity and so forth) that affect the enzyme structure or the rate-limiting step [5]. In tomato juice, the PME activity was 1.62 times that of the control as was found under 600 Mpa/20 °C/10 min [6]. 

Recently some studies have investigated persimmon treated by HPP. For example, Rodriguez et al. showed that HPP under 400 Mpa/1 min performed on persimmon puree could eliminate microbial counts and show no regrowth during storage, and that there was also good physicochemical attributes, phenolic compounds, and antioxidant capacity of the puree which was stable during storage. The HPP, however, was not able to effectively inhibit enzyme activity [7]. Kumari et al. also observed that persimmon pulp treated by HPP under 20 °C/400 Mpa/5 min had a reduced total plate count and yeast mold count [8]. Moreover, José et al. studied HPP under 200 Mpa for improved carotenoid extractability and tannin polymerization in order to improve the functionality and taste of fresh-cut persimmon [9]. Similar results were studied by de Ancos et al. [10], Lucía et al. [11] and Cano et al. [12]. Furthermore, Vázquez et al. explained that HPP affected the structure of persimmon significantly, which influences the integrity of cell walls and membranes, so as to improve the extractability of carotenoid and soluble tannin [13]. Additionally, Hernández-Carrión et al. compared new milk-based persimmon beverages treated by HPP and pasteurization and found that HPP was good for releasing carotenoids from the fruit matrix, precipitating tannins and rheological properties. The untreated and pasteurized persimmon milk-based beverages had either a gel-like structure or became separated out [14]. In conclusion, as a novel and important non-thermal processing, a HPP treatment could guarantee the safety, nutrition and sensory attributes of persimmon and its products, and it has the potential to activate enzymes which are good for persimmon processing; however, no one has previously studied a HPP treatment for the juice yield of persimmon or the quality of persimmon juice. Therefore, this work’s purpose was to design a processing technology for persimmon juice which could solve the low juice yield problem and avoid exogenous pectinase from being added. Firstly, three persimmon cultivars were compared and one of them was selected as the appropriate cultivar for persimmon juice processing. Secondly, the impacts of the HPP pre-treatment on the juice yield and pectinases in persimmon pulp were studied to increase the yield and quality of the juice. Finally, the comparison of HPP and TP as the microbial inactivation procedure was completed and the microorganisms, enzymes, color differences and other physicochemical properties (e.g., pH, total soluble solids, clarity, total phenols, ascorbic acid, soluble tannin and antioxidant capacity) in persimmon juice were studied.

## 2. Materials and Methods

### 2.1. Materials and Chemicals

**Figure 1:**
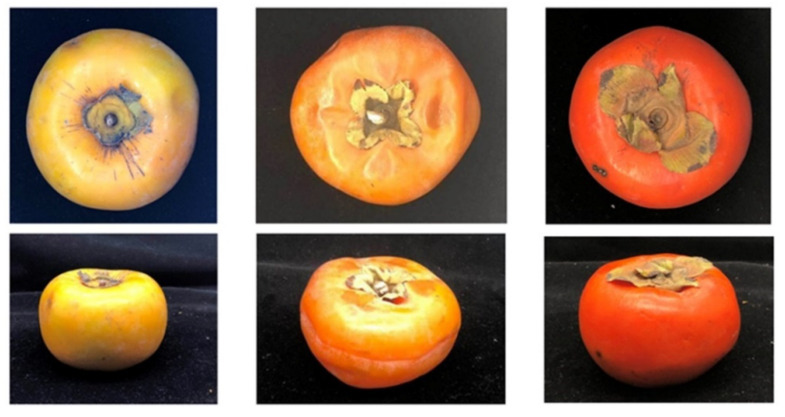
Persimmon (*Diospyros kaki* L.) cultivars Gongcheng, Mopan and Yangfeng (left to right).

Fresh and mature persimmons of three cultivars were purchased in Beijing market (shown in Figure 1). The “Gongcheng” cultivar was yellowish and crispy, which was harvested in Guangxi, China; the “Mopan” cultivar was orange-red with a thick peel and soft flesh, which was harvested in Beijing, China; and the “Yangfeng” cultivar was a deep orange-red with moderate hardness, which was harvested in Shanxi, China.

The 2,2-diphenyl-1-picrylhydrazyl (DPPH), (±)-6-hydroxy-2,5,7,8-tetramethyl-chroman-2-carboxylic acid (Trolox), and 2,4,6-tri-2-pyridyl-1,3,5-triazine (TPTZ) were obtained from Sigma Aldrich (St. Louis, USA). High performance liquid chromatography (HPLC)-grade acetonitrile, methanol and formic acid were purchased from Thermo Fisher Scientific (MA, USA). Other chemicals were obtained from Solarbio (Beijing, China).

### 2.2. Preparation of Persimmon Pulp and Juice

The persimmons were washed with tap water, cut into pieces and pulped. The persimmon pulp was kept at 20 ± 1 °C for 3 h and then centrifuged under 12,000× *g*/10 min/4 °C (CR21GIII, Hitachi Limited, Japan). The supernatant was gathered as persimmon juice.

### 2.3. HPP and TP Treatments of Persimmon Pulp and Juice

Pre-HPP (100, 200, 300 and 400 MPa for 10 min; 300 MPa for 2, 5, 8 and 10 min) and HPP (550 MPa for 5 min) treatments of the persimmon pulp and persimmon juice were conducted with a high pressure processing unit (CQC30L-600; Suyuan Zhongtian Scientific Co., Ltd., Beijing, China) at room temperature (20 °C). The holding time of the HPP did not include the time of pressurization and depressurization. 

The TP treatments were conducted by keeping the persimmon juice (in 10 mL polyethylene terephthalate bottles) at 95 °C for 5 min in a water bath and then cooling to 20 °C.

**Figure 2:**
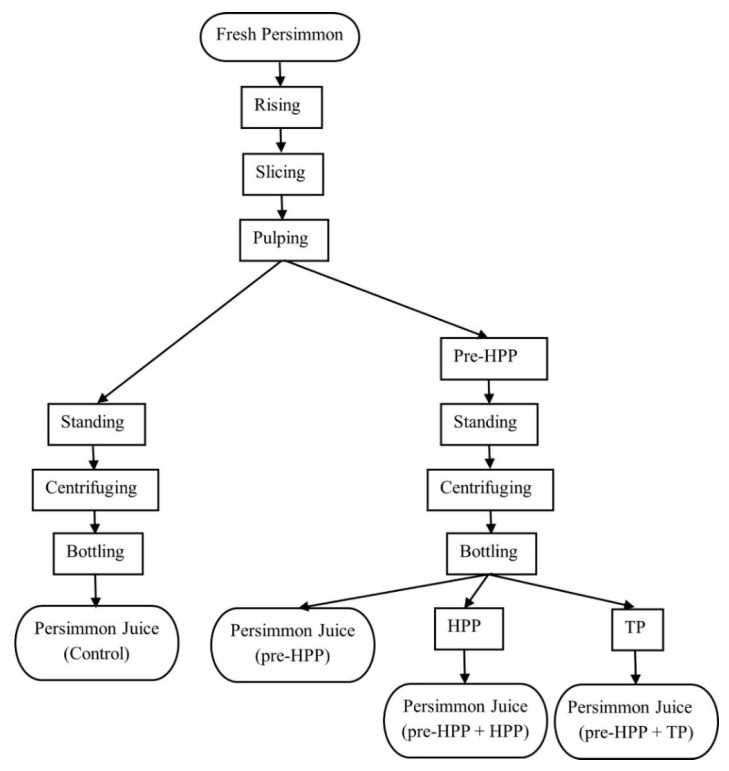
Flow chart of persimmon juice processing.

The flow chart of the persimmon juice processing in this study is shown in Figure 2.

### 2.4. Determination of pH

The pH value of the persimmon pulp and juice were measured at 25 ± 1 °C, with a Thermo Orion 868 pH meter (Thermo Fisher Scientific, Inc., Waltham, MA, USA).

### 2.5. Determination of Total Soluble Solids (TSS)

The TSS of the persimmon pulp and juice were determined at 25 ± 1 °C by a WAY-2S Digital Abbe Refractometer (Shanghai Precision & Scientific Instrument Co., Shanghai, China), and the results were reported as oBrix.

### 2.6. Determination of Juice Yield

The persimmon pulp was centrifuged under 12,000× *g*/10 min/4 °C. Then, the supernatant was collected as persimmon juice. The juice yield was calculated using Equation (1), where *M_juice_* is the weight of the persimmon juice and *M_pulp_* is the weight of the persimmon pulp:

Juice yield (%) = *M_juice_*/*M_pulp_* (1)

### 2.7. Determination of Total Phenols

The total phenols of the persimmon pulp and juice were measured by Folin–Ciocalteu as described by Kim with some modifications [15]. A 2 mL amount of persimmon juice or pulp was first added to a vessel with 2 mL methanol (80%) and then extracted for 20 min with an ultrasonic machine. Then, the mixture was centrifuged under 12,000× *g*/10 min/4 °C. The supernatant of 0.4 mL and 2 mL Folin–Ciocalteu reagent (both previously diluted ten-fold using distilled water) were mixed with 1.8 mL of sodium carbonate solution (7.5%) and then left for 1 h in the dark at room temperature. After that, the mixture needed to be measured immediately at 765 nm using a spectrophotometer (UV-1800, UNICO, Shanghai, China) and the results were reported as a mg of gallic acid equivalent (GAE) per 100 g juice.

### 2.8. Determination of Ascorbic Acid (AA)

The ascorbic acid levels of the persimmon pulp and juice were determined by a HPLC (LC-20AT, Shimadzu, Kyoto, Japan) equipped with a pump, a UV–Vis detector and a data acquisition system. The chromatographic column was a Venusil XBP C18 column (4.6 × 250 mm, 5 μm) from the Angela Company, and the column temperature was 30 °C. The persimmon pulp or juice (5 g) was mixed with a 1% formic acid solution (5 mL). After extraction for 2 h at 4 °C and centrifugation under 12,000× *g*/10 min/4 °C, the supernatant was filtered through a 0.45 μm membrane and analyzed by HPLC. The flow rate was 1 mL/min, the mobile phase was 10 mL of acetonitrile in a 90 mL formic acid solution (1%), the sample injection volume was 20 μL and detection was set at 254 nm [16].

### 2.9. Sensory Evaluation

Sensory evaluation of persimmon juices from the 3 different cultivars were carried out by ten panelists and were conducted following the protocol in NY 82.2-1988 [17] with some modifications. The persimmon juice samples were placed in small plastic cups (50 mL juice per cup) and identified with random three-digit codes. All samples were evaluated at about 18 °C. The panelists scored the samples for color, flavor, mouthfeel, appearance, and acceptability. Each attribute was scored from 1 to 4. Higher numbers represented a stronger performance. The results were reported as the total score of 5 attributes for the persimmon juice from each cultivar.

The sensory evaluation was carried out under low intensity light conditions to guarantee correct color and appearance evaluation. For acceptability, the panelists first evaluated the flavor and then tasted samples to evaluate the mouthfeel. It should be noted that the panelists were instructed to cleanse their palate with taste-free water (Wahaha, Hangzhou, China) after evaluating each sample.

### 2.10. Determination of Pectin Methylesterase (PME) Activity

The PME activity of the persimmon juice and pulp were determined by measuring the release of acid at pH 7.5/30 °C, as described by Bi with some modifications [18]. Persimmon pulp or juice (5 g) mixed with a 0.02 M Tris-HCl buffer (10 mL, pH 7.5, containing 0.1 M NaCl) was kept for 12 h at 4 °C, and then centrifuged at 12,000× *g* for 10 min at 4 °C. The PME activity was detected with an automatic potentiometric titrator (751 GPD Titrino, Metrohm, Herisau, Switzerland). The supernatant (5 mL) was added into a 60 mL 1% pectin solution *(w/v*, pH 7.5, containing 0.1 M NaCl). NaOH (0.05 M) was automatically added to keep the solution pH at 7.5. The slope of the linear portion of the graph of *V_NaOH_* (in milliliters) versus *t* (in minutes) was obtained to calculate the PME activity using the following Equation (2), where *C_NaOH_* is the NaOH concentration (0.05 M), and *V_sample_* is the sample volume (5 mL of the supernatant). The results were expressed as PME activity units (mmoL·L^−1^·min^−1^), where one unit equals 1 mmoL NaOH consumed per liter sample per minute:

PME activity = Slope × *C_NaOH_/V_sample_* (2)

### 2.11. Determination of Polygalacturonase (PG) Activity

The PG activity of the persimmon pulp or juice were determined as the method described by Amnuaysin with some modifications [19]. Persimmon pulp or juice (2 g) was mixed with 6% NaCl (6 mL, containing 0.6% EDTA and 1% PVP) and centrifuged under 12,000× *g*/20 min/4 °C. After that, 0.1 mL supernatant (previously diluted ten-fold using distilled water) was added into a 1.0 mL pectin solution (0.5%, *w/v*) and kept at 37 °C for 30 min. After adding 0.9 mL DNS reagent, the samples were kept in a boiling water bath for 5 min and then measured at 540 nm using a UV-1800 spectrophotometer (UNICO, Shanghai, China). The results were expressed as PG activity units (U·g^−1^), where one unit equals 1 g free galacturonic acid produced by the decomposition of pectin at 37 °C per gram of fresh sample per minute.

### 2.12. Microbiological Analysis

To detect natural microorganisms in the persimmon juice, the total plate count method was used [20]. Samples were diluted with a sterile 0.9% NaCl solution one by one, followed by plating in a 1.0 mL dilution into duplicate plates of appropriate agar. Plate count agar (Beijing Solarbio Science & technology Co. Ltd., Beijing, China) was used for the total aerobic bacteria (TAB) counting after incubation at 37 °C for 48 ± 2 h. Rose Bengal agar (Beijing Solarbio Life Science Co. Ltd., Beijing, China) was used for the yeasts and molds (Y&M) counting after incubation at 28 °C for 72 ± 2 h. The colonies were counted and log10 *N* was used for describing the inactivation effect, where *N* is the number of microorganisms.

### 2.13. Determination of Water-Soluble Pectin

The extraction and measurements of soluble pectin in the persimmon juice were described by Rose et al. with some modifications [21]. Persimmon juice (10 g) was added to 95% ethanol (30 mL), kept in a boiling water bath for 20 min, and then filtered with a G4 sand core funnel. After being dried at 35 °C for 24 h, the powder (20 mg) was added to 7.5 mL of distilled water and centrifuged under 12,000× *g*/10 min. The supernatant (1 mL) was mixed with concentrated sulfuric acid (10 mL) and kept at 85 °C for 25 min. After adding 0.8 mL Carbazole-ethanol (0.15%), the solution was measured at 540 nm using a UV-1800 spectrophotometer (UNICO, Shanghai, China). The results were expressed as micrograms of galacturonic acid per gram of fresh weight.

### 2.14. Determination of Soluble Tannin

The soluble tannin of the persimmon juice was determined by a HPLC (LC-20AT, Shimadzu, Japan) equipped with a pump, a UV–Vis detector and a data acquisition system. The chromatographic column was a Venusil XBP C18 column (4.6 × 250 mm, 5 μm) from the Angela Company, and the column temperature was 30 °C. The persimmon juice (5 g) was mixed with ultrapure water (5 mL). After extraction for 30 min at 50 °C and centrifugation under 12,000× *g*/10 min/4 °C, the supernatant was filtered through a 0.45 μm membrane and analyzed by HPLC. The flow rate was 1 mL/min, the mobile phase was 20 mL of ultrapure water in 80 mL methanol, the sample injection volume was 20 μL and detection was set at 275 nm [22].

### 2.15. Determination of Antioxidant Activity

#### 2.15.1. DPPH Radical Scavenging Assay (DPPH)

This assay was based on measuring the scavenging ability of antioxidants to the stable radical •DPPH [23]. The extract (200 μL) was mixed with 0.14 mM methanolic •DPPH solution (4 mL). A control reaction was prepared with 200 μL of methanol mixed with 4 mL of a 0.14 mM methanolic •DPPH solution. The samples were kept in the dark for 45 min at room temperature before measuring the decrease in the absorption at 517 nm with a UV-1800 spectrophotometer (UNICO, Shanghai, China). Radical scavenging activity was calculated by Equation (3), where *A*_0_ is the absorbance of the control and *A_S_* is the absorbance in the presence of the persimmon extract. The results were expressed as milligrams of Trolox equivalents per 100 mL of persimmon juice (TE/100 mL):

Radical scavenging activity (%) = (*A_S_* − *A*_0_)/*A*_0_ × 100 (3)

#### 2.15.2. Ferric Reducing/Antioxidant Power Assay (FRAP)

This assay referred to Aljadi and Kamaruddin with some modifications [24]. The freshly prepared FRAP solution contained 0.3 mol/L acetate buffer (25 mL, pH 3.6), 2.5 mL of 10 mmoL/L TPTZ (dissolved in 40 mmoL/L HCl), and 20 mmoL/L ferric chloride (2.5 mL).

The FRAP solution (4 mL) was mixed with the extract (100 μL), and the mixture was kept under 37 °C/10 min. The ferric reducing ability was measured by using the absorbance at 593 nm using a UV-1800 spectrophotometer (UNICO, Shanghai, China). The results were expressed as milligrams TE/100 mL.

### 2.16. Determination of PPO and POD Activity

The enzymes were extracted and determined according to Zhang et al. with some modifications [25]. For the enzyme extraction, 2.5 g of persimmon juice was mixed with a 5 mL buffer (0.2 M, pH 6.5, phosphate buffer with 4% (*w/v*) PVPP). After standing at 4 °C for 1 h, the mixture was centrifuged under 12000 g/10 min/4 °C, and the supernatant was collected and analyzed for enzyme activity. The PPO and POD activity were determined by a Spark 10 M microplate spectrophotometer (Tecan, Mennendorf, Switzerland) based on the absorbances of 420 nm and 470 nm, respectively. The reaction mixture for the PPO was 10 μL of extract and 190 μL of catechol (0.1 M) in a phosphate buffer (0.2 M, pH 6) solution. The reaction mixture for the POD was 190 μL of guaiacol (1.0%, *m/v*, dissolved in a 0.2 M phosphate buffer, pH 6), H_2_O_2_ (1%, *m/v*), and 10 μL of the diluent extract.

### 2.17. Color Assessment

The color assessment was carried out at 25 ± 2 °C using a color difference meter (Color Quest XE, Hunter Associates Laboratory Inc., Reston, VA, USA). The measured parameters were L* for lightness, a* for redness, and b* for yellowness. In addition, the total color difference (Δ*E*) was calculated using Equation (4), where *L*_0_*, *a*_0_*, and *b*_0_* were the control values for the untreated juice:

Δ*E* = [(L* – L_0_*)^2^ + (a* – *a*_0_*)^2^ + (b* – *b*_0_*)^2^]^1/2^ (4)

### 2.18. Determination of Browning Degree (BD)

The determination of BD was described by Meydav with some modifications [26]. Persimmon juice (5 mL) was mixed with ethanol (5 mL) and centrifuged under 12,000× *g*/10 min/4 °C. The BD was determined by measuring the absorbance of the supernatant at 420 nm using a UV-1800 spectrophotometer (UNICO, Shanghai, China).

### 2.19. Determination of Clarity

The clarity of the persimmon juice was determined by measuring the transmittance of juice at 625 nm using a UV-1800 spectrophotometer (UNICO, Shanghai, China) [27].

### 2.20. Statistical Analysis

All experiments were made in triplicate. The data were analyzed using the Statistical Program for Social Sciences (SPSS 23, Chicago, IL, USA) software for the analysis of variance and Duncan’s test. The significance was established at *p* < 0.05.

## 3. Results and Discussion

### 3.1. Differences on Physical and Chemical Indexes of Three Persimmon Cultivars

**Figure 3:**
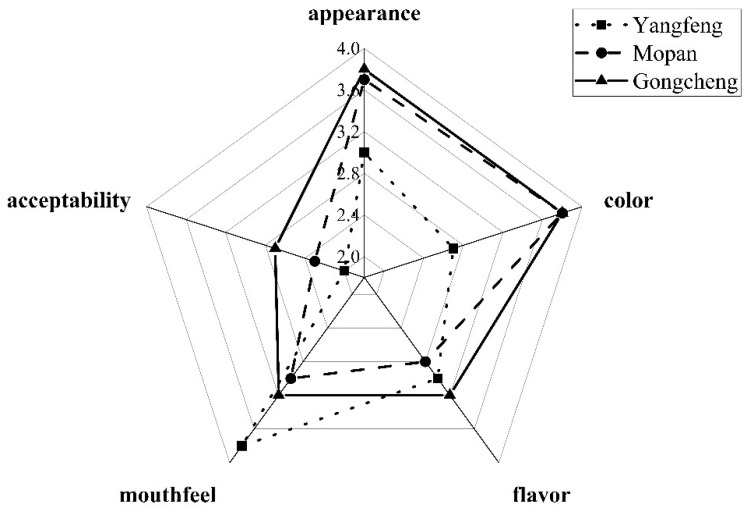
Sensory evaluation of juice from three persimmon cultivars.

In order to select a suitable persimmon cultivar for juicing, some basic indexes of three persimmon cultivars including “Gongcheng” from Guangxi, “Mopan” from Beijing and “Yangfeng” from Shanxi were compared in Table 1. The most important index for juicing is the juice yield, which was 41.03% in the Gongcheng persimmon, and which was the highest of the three cultivars. The total phenol content in the “Gongcheng” was significantly higher than that in the other two cultivars, reaching 6.78 mg GAE/100 g and the content of AA in the “Gongcheng” was in the middle range of the three. Using a sensory score to evaluate the different cultivars (details shown in Figure 3), “Gongcheng” received the highest score as shown in Table 1. After synthetical analysis of the juice yield and the nutrition content, the “Gongcheng” was recommended to be used as the raw material for the persimmon juice in the following study.

### 3.2. Effects of Different Pre-HPP Treatment Pressure and Time on Juice Yield, PME and PG Activity of Persimmon Pulp

**Figure 4:**
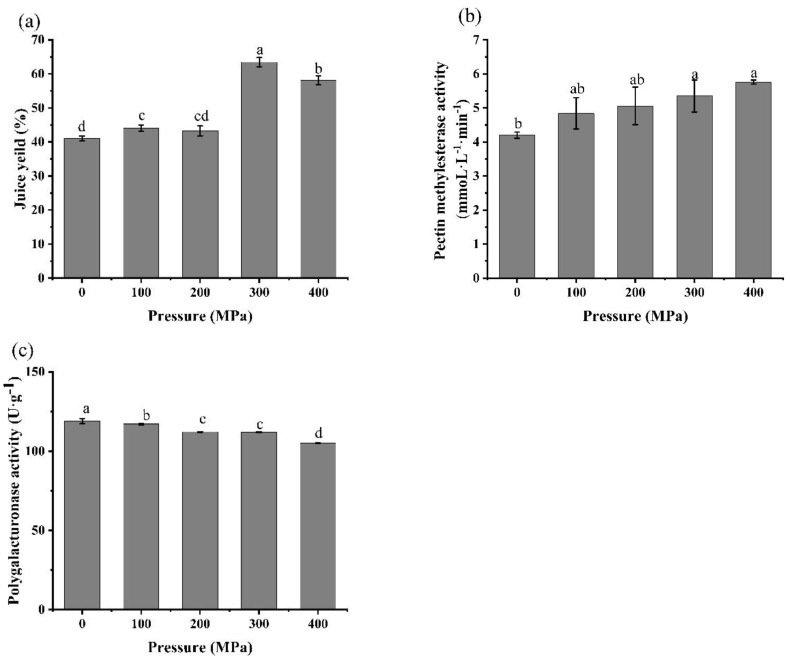
Effects of different pre−HPP treatment pressure on (**a**) juice yield, (**b**) pectin methylesterase activity, and (**c**) polygalacturonase activity in “Gongcheng” persimmon pulp for 10 min. (Different letters in the same graphic indicate a significant difference (*p* < 0.05).).

As shown in Figure 4, the pre-treatment of the persimmon pulp by HPP could increase the juice yield and PME activity, but slightly decrease the PG activity. Compared with the pre-HPP treatment of 100, 200, 300 and 400 MPa for 10 min, the juice yield maximum increased by 22.42% after 300 MPa/10 min. For the juice yield relative enzyme, the activity of the PME and PG was increased by 27.4% and slightly decreased by 5.8% individually after 300 MPa/10 min pre-treatment. A similar phenomenon was also reported elsewhere that the PME in tomato juice was activated by 300 MPa/10 min/25 °C with an almost 50% increase and the PG was decreased by approximately 15% [28]. Liu et al. reported that PME activity was activated by 10–40% when mango pulp was treated with 300–500 MPa for 5 min, while the residual activity of mango pulp treated with 110 °C/8.6 s was only 3% of the control group [20].

The activation of the PME by pre-HPP could be due to an increase of the de-esterification reaction rate by volume being reduced resulting from electrostriction [5]. Moreover, the HPP treatment could affect the tertiary and quaternary structure of the enzyme by modifying the enzyme’s electrostatic, hydrophobic interaction and hydrogen bonding, exposing more active sites or increasing the size of existing active sites [29], thereby promoting endogenous PME demethylation esterification. The changes of the substrate molecules induced by HPP was also suggested as a reason for the increased rate of PME catalyzed reactions, e.g., the disruption of membranes, releasing the membrane-bound PME and promoting the binding of enzymes and substrates [28]. Crelier et al. reported that the PG in tomatoes had a certain stability under high pressure, and its thermal stability was stronger than that of the PME—heat treatment alone or HPP treatment at room temperature could not inactivate the enzyme effectively—which was consistent with the experimental results [30,31].

The increase in PME activity had positive influence on the juice yield, as the PME could break down the pectin molecules so that the structure-broken cells released more juice. In detail, the pectin molecule is a polysaccharide chain formed by the linear polymers of D-galacturonic acid with different esterification degrees with α-1,4 glycosidic linkages. It has a very high colloidal stability and water-binding capacity, which makes it difficult for fruit juice to flow out during the process of fruit extrusion [32]. The PME de-esterifies the methyl groups on the galacturonic acid backbone of pectin which enhances the juice yield [33]. Moreover, the PG catalyzes the hydrolytic cleavage of the glycosidic α-D-(1–4) bonds in the pectic acid and leads to a viscosity decrease, hence the juice yield increased as well. Low ester pectin produced by the PME de-esterifying would also combine with Ca^2+^ and form the Ca^2+^ pectate gels which influences the juice quality; however, the retained PG could compete with the Ca^2+^ inducing the Ca^2+^ pectate gels and therefore guarantee good quality of the persimmon juice.

**Figure 5:**
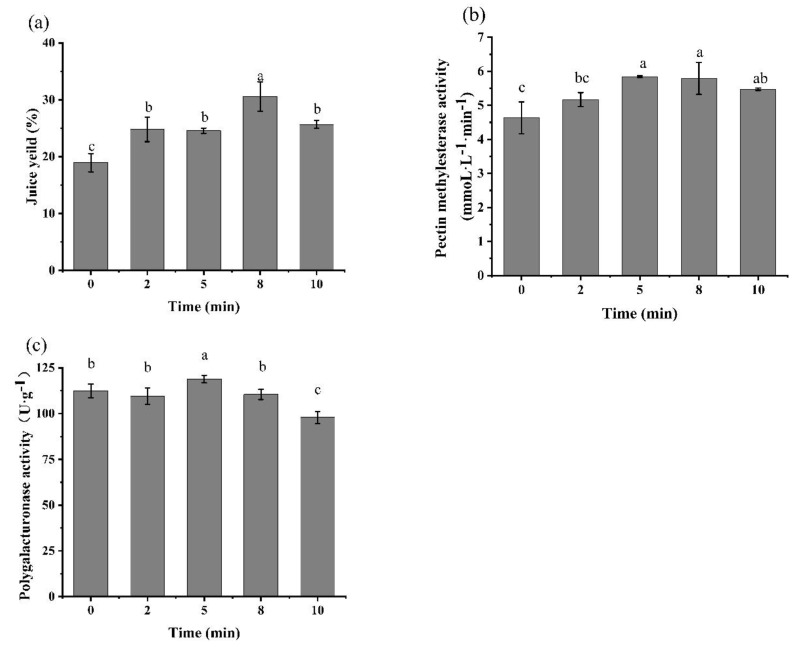
Effects of different pre−HPP treatment times on (**a**) juice yield, (**b**) pectin methylesterase activity, and (**c**) polygalacturonase activity in persimmon pulp under 300 MPa. (Different letters in the same graphic indicate a significant difference (*p* < 0.05).).

As the activation of the PME and retention of the PG by the pre-HPP treatment at 300 MPa could lead to an increased yield in the persimmon juice, different treatment times at 300 MPa were compared in Figure 5. The juice yield of the persimmon pulp treated with 300 MPa for 2, 5, 8 and 10 min, were all significantly higher than that of the control. After 300 MPa/8 min, the persimmon juice yield was the highest, which was 1.6 times higher than that of the control group. The PME activity of the persimmon pulp treated with 300 MPa for 5, 8 and 10 min, was significantly increased and the PME in samples treated by 300 MPa/5 min was the highest with no significant difference compared with the 8 and 10 min samples, which was nearly 26% higher than that of the control. The activity of PG in the persimmon pulp did not change after 300 MPa for 2 and 8 min, while it slightly increased after 5 min and decreased after 10 min. Since the persimmon pulp treated by pre-HPP (300 MPa/8 min) achieved the highest juice yield, and increased PME activity and maintained PG activity, a pre-HPP of 300 MPa/8 min was recommended to be used in the pre-treatment of persimmon juice.

### 3.3. Effects of HPP and TP on Microorganism, pH and TSS of Persimmon Juice

As shown in Table 2, the initial total aerobic bacteria (TAB) in the persimmon juice were 5.61 lg CFU/mL, and after pre-HPP, it was still more than 5 lg CFU/mL. The TAB decreased to 1.29 lg CFU/mL after pre-HPP plus HPP (550 MPa/5 min), which meets the standard of the National Food Safety Standard-Beverage GB 7101-2015 in China. In the pre-HPP plus TP (95 °C/5 min) samples, the TAB was not detected. No yeast and molds were detected in all the samples treated by the pre-HPP plus HPP or pre-HPP plus TP. The values of pH and TSS in the pre-HPP (300 MPa/8 min) treated juices were unchanged; however, in samples of the pre-HPP plus HPP and pre-HPP plus TP, the pH value decreased significantly. The reduction in pH was explained by the degradation of tissue when the samples were treated with a higher pressure again [34].

### 3.4. Effects of HPP and TP on PME, PG, and Water-Soluble Pectin in Persimmon Juice

**Figure 6:**
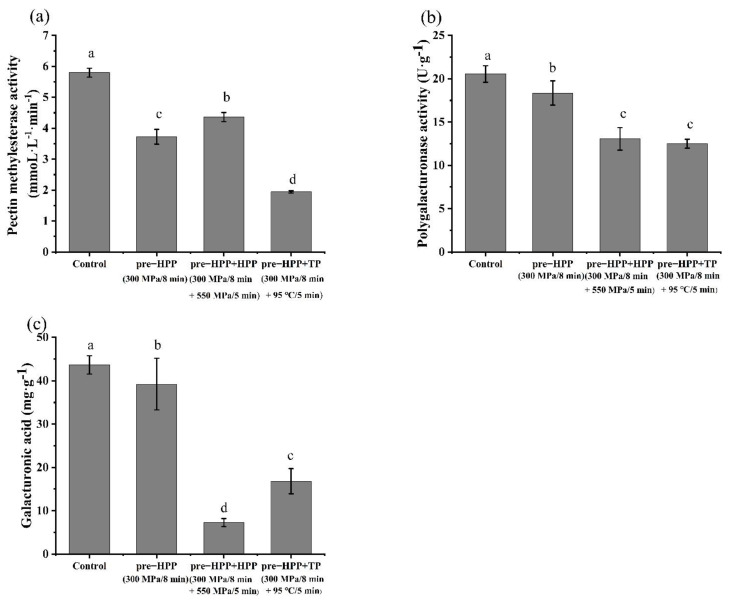
Effects of different treatments on (**a**) pectin methylesterase activity, (**b**) polygalacturonase activity, and (**c**) galacturonic acid in persimmon juice. (Different letters in the same graphic indicate a significant difference (*p* < 0.05).).

In Figure 6a, after a pre-HPP treatment at 300 MPa/8 min, the PME activity in the persimmon juice significantly decreased from 5.80 ± 0.14 mmoL·L^−1^·min^−1^ to 3.72 ± 0.24 mmoL·L^−1^·min^−1^, which is inconsistent with a previous study that observed PME activity in persimmon pulp significantly increased from 4.20 ± 0.09 mmoL·L^−1^·min^−1^ to 5.35 ± 0.47 mmoL·L^−1^·min^−1^ (as shown in Figure 4b). The reason for this inconsistency with the previous study was probably due to the different initial status of the PME in the persimmon raw material. A higher maturity of persimmon was used in this part of the study compared with the previous study, with most of the PME existing as the unbound stage, hence the initial PME activity was higher than the raw material with a lower maturity. Based on previous discussion, activation of the PME by the pre-HPP was probably mainly due to the HPP releasing the membrane-bound PME and promoting the binding of enzymes and substrates. According to the results in this study, it is likely difficult for the extra membrane-bound PME in higher mature persimmons to dissolve in the system after a pre-HPP treatment. On the other hand, HPP may have changed the construction of the PME, which caused the decrease in PME activity in this study. Although no increase in PME activity was found in this study, the juice yield still increased (Table 2), indicating that the acceleration of the PME and PG reaction by the HPP played an important role in increasing the juice yield.

After the pre-HPP plus HPP, PME activity in the persimmon juice significantly increased by 17.2% compared with the pre-HPP treated samples, which was probably due to the further activation of PME in the persimmon juice after second round of HPP, whereas only 52.15% was left after thermal processing. Compared with the control group, the activity of the PG in the persimmon juice decreased by 10.6% significantly after the pre-HPP (300 MPa/8 min), and all decreased to around 60% of the control after the pre-HPP plus HPP and pre-HPP plus TP, indicating that all of the three treatments could well retain the activity of the PG which is good for the juice yield.

After the pre-HPP, pre-HPP plus HPP and pre-HPP plus TP, the soluble pectin content in the persimmon juice all decreased significantly, which was possibly due to the enzymatic hydrolysis of the pectin or the formation of a pectin–tannin complex. In the pre-HPP plus HPP treated samples, the pectin was the lowest and decreased to 16.66% of the control group, with a higher PME activity than the other two treatments. This suggests that the higher enzyme activity favors the hydrolysis of pectin. The TP could trigger the degradation of the pectin by β-elimination, especially when the pH is higher than 4.5 [35].

### 3.5. Effects of HPP and TP on Total Phenols, Ascorbic Acid, Antioxidant Capacity and Soluble Tannin in Persimmon Juice

**Figure 7:**
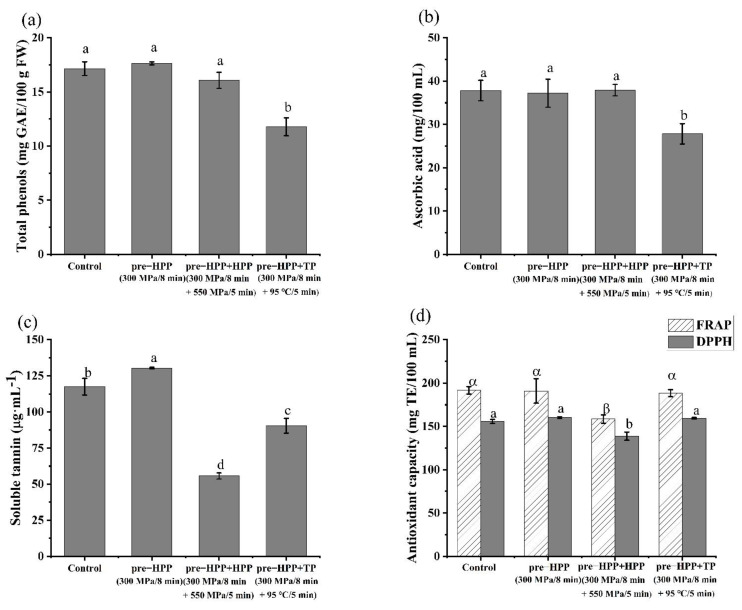
Effects of different treatments on (**a**) total phenols, (**b**) ascorbic acid, (**c**) soluble tannin, and (**d**) antioxidant capacity in persimmon juice. (Different letters in the same graphic indicate a significant difference (*p* < 0.05).).

HPP could well retain the content of total phenols and AA in persimmon juice. After pre-HPP and pre-HPP plus HPP, the total phenol content of the persimmon juice did not change significantly (Figure 7a), while in the pre-HPP plus TP treated samples, it was decreased significantly to 66.48% of the control group. The samples treated by the pre-HPP and pre-HPP plus HPP showed no significant difference in AA content, while in the pre-HPP plus TP treated samples, the AA content decreased by 17.02% (Figure 7b). A large number of studies have reported that heat treatment has a destructive effect on AA, while HPP treatment could maintain the AA content well. Patras et al. showed that the levels of phenols in strawberry and blackberry purées treated at 600 MPa increased significantly as compared to unprocessed purée [16], and that a HPP at a different pressure did not cause any significant change in AA, while following thermal processing the AA degradation was 21% as compared to unprocessed purées.

Tannin is a phenolic substance, which can precipitate alkaloids, gelatin and other proteins. It has the function of antioxidation, free radical capture and bacteriostasis [36]. Tannin in fruit juice can protect color and improve clarity, but it can also bring some undesirable effects, such as an astringent taste, and can precipitate easily during storage of the juice. Persimmon tannin is a kind of polymer composed of catechin, catechin-3-galic acid, gallocatechin, and gallocatechin-3-galic acid. The degree of polymer polymerization directly affects the astringency intensity of persimmon. Compared with the stage of aggregation, the water-soluble tannin is the main factor that causes an astringent taste. In Figure 7c, the soluble tannin content in the persimmon juice significantly increased by 10.84% after the pre-HPP which may have been caused by the HPP changing the permeability of the cell membrane and by more phenolic substances, such as tannin, being released. Moreover, the HPP was able to break the non-covalent bonds between the tannins and other macromolecular compounds, releasing more soluble tannins. In the samples treated by the pre-HPP plus HPP, the soluble tannin content significantly decreased by 57.28% compared to the pre-HPP treated samples, and was 38.48% lower than the thermal processing samples, indicating that a second round of HPP could decrease the astringency intensity of persimmon juice. Vázquez-Gutiérrez et al. reported that the soluble tannin content decreased significantly after HPP (200 MPa/6 min/37 °C) in persimmon [37]. This phenomenon was explained by the precipitation of tannin, a result of the disruption of the cell wall and membrane with the soluble materials in the cell flowing out into the intercellular space, such as pectin and protein, which could then bind the soluble tannin [13]. The decrease in tannin content after thermal processing may be due to the decomposition of tannin at high temperature, or the formation of the tannin compound [38].

Two methods of DPPH and FRAP were used to evaluate the antioxidant capacity of the persimmon juice (Figure 7d). The changes in the antioxidant capacity of the persimmon juice were positively correlated to the content changes of total phenols, AA and soluble tannin [39]. Among the three different evaluated stages, a decrease in the antioxidant capacity was only found in the pre-HPP plus HPP treated samples, which was probably due to the second round of HPP accelerating the combination of water-soluble tannin with the protein, pectin and so forth, in order to decrease the water-soluble tannin.

### 3.6. Effects of HPP and TP on the Polyphenol Oxidase Activity, Peroxidase Activity and Color of the Persimmon Juice

**Figure 8:**
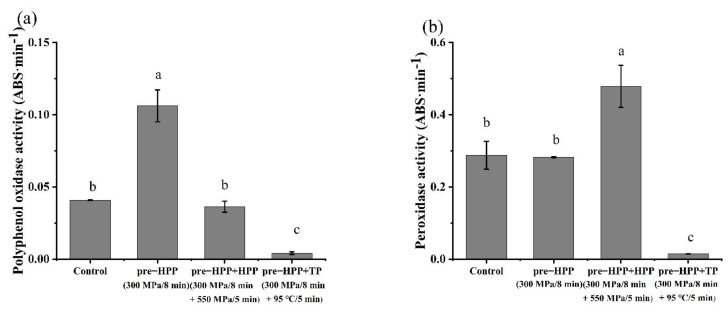
Effects of different treatments on (**a**) polyphenol oxidase activity and (**b**) peroxidase activity in persimmon juice. (Different letters in the same graphic indicate a significant difference (*p* < 0.05).).

The existence of polyphenol oxidase (PPO) in fruit juice is the main reason for enzymatic browning of fruit juice. The PPO can catalyze hydroxylation of free phenolic acid and dehydrogenation of hydroxy phenol to quinone in fruits and vegetables. Quinone condenses itself in fruits and vegetables or reacts with proteins in cells to produce brown pigments or melanin, which results in changes in the appearance and color of fruit juice [40]. The PPO activity increased significantly to 2.65 times of the control group after pre-HPP (300 MPa/8 min) (Figure 8a). This result is consistent with the significant increase in enzyme activity in strawberry juice treated with 300 MPa as reported by Liu et al. [41]. This activation was hypothesized to be a result of the release of enzymes from the membrane or enzyme–inhibitor complex, or a limited conformation change. After HPP (550 MPa/5 min), the PPO activity was restored to the same level of the control group. This could be explained by the processing with a higher pressure more optimal for inactivating the PPO or by the changes induced by the pre-HPP that were reversible [42]. TP treatment significantly reduced vitality to 10.6% of the control group through destructing the enzyme structure. 

Peroxidase (POD) is also related to the browning of fruit juice, and the reaction products catalyzed by POD will affect the taste and flavor of fruit juice [43]. The POD activity did not change significantly after pre-HPP (300 MPa/8 min), but it was significantly activated after the HPP (550 MPa/5 min), where its activity was 1.66 times higher than that of the control group. Similar to PME, it is speculated that POD activation is related to the release of enzymes from the membrane or enzyme–inhibitor complex after HPP. Similar to PPO, TP also significantly reduced POD activity to 3.58% of the control group.

According to Table 3, the pre-HPP did not induce an obvious color change in the persimmon juice with a Δ*E* of 0.35 (Δ*E* < 2, the color change is invisible to the naked eye). Additionally, the browning degree decreased significantly. The color change of the persimmon juice treated with pre-HPP plus HPP was 2.68, slightly noticeable (2 < Δ*E* < 3), but the changes of L*, a*, b* as well as the browning degree were not significant. This might suggest that the enzymatic browning of the persimmon juice was affected by both the PPO activity and the phenolic content, especially the soluble tannin [44]. Although the pre-HPP samples had a higher PPO activity, they also contained the highest values of soluble tannin. With the pre-HPP plus TP samples, the color change was 15.38, and this was obvious (Δ*E* > 12, can be considered as different colors), with significant changes in L* and b*. The HPP treatment had less effect on color than the TP treatment. The clarity of the pre-HPP plus HPP treated samples showed no significant difference from the control group. After the pre-HPP plus TP treatment, the persimmon juice produced a large amount of white suspended matter, resulting in the clarity of the juice being reduced to less than 1%. It was believed that the turbidity of the persimmon juice is caused by the combination of tannin with protein or other macromolecule substances, or by the coagulation and precipitation of colloids in the persimmon juice after thermal treatment [45].

## 4. Conclusions

“Gongcheng” was selected as the appropriate cultivar for the processing of persimmon juice, owing to its high juice yield, content of total phenols and score in the sensory evaluation. With endogenous PME activated by 25.03%, the juice yield of the persimmon pulp treated with pre-HPP (300 MPa/8 min) and standing at 20 °C for 3 h, significantly increased by 60%, instead of adding an exogenous pectinase as with traditional juice processing technology. Both the pre-HPP plus HPP (550 MPa/5 min) and pre-HPP plus TP (95 °C/5 min) treatments inactivated the TAB and Y&M below 2 log units. The color, clarity, AA and phenols were better retained after pre-HPP plus HPP and with a lower content of soluble tannin than the treatment with pre-HPP plus TP. The treatment with pre-HPP plus HPP could effectively increase the juice yield, increase the shelf life, maintain a good quality of persimmon juice and avoid re-astringency and turbidity.

## Figures and Tables

**Table 1:** Physical and chemical indexes of three different persimmon cultivars.

	Gongcheng (Guangxi)	Mopan (Beijing)	Yangfeng (Shanxi)
Juice yield (%)	41.03 ± 0.75 a	21.84 ± 2.41 b	10.27 ± 0.47 c
pH	5.83 ± 0.01 c	6.00 ± 0.01 b	6.28 ± 0.02 a
TSS (oBrix)	14.60 ± 0.10 c	15.00 ± 0.10 b	17.30 ± 0.10 a
Total phenols (mg GAE/100 g FW)	6.78 ± 0.33 a	2.78 ± 0.28 b	1.93 ± 0.17 c
Ascorbic acid (mg/100 g FW)	28.32 ± 1.42 b	18.25 ± 2.79 c	97.53 ± 3.40 a
Sensory evaluation	16.70	15.60	14.50

All data were the means ± SD, *n* = 3. Different letters in the same row indicate a significant difference (*p* < 0.05). TSS: total soluble solids.

**Table 2:** Effects of different treatments on microbial, pH, TSS and juice yield levels of persimmon juice.

Treatments	Control	Pre-HPP (300 MPa/8 Min)	Pre-HPP + HPP (300 MPa/8 min + 550 MPa/5 Min)	Pre-HPP + TP (300 MPa/8 min + 95 °C/5 Min)
TAB (lg CFU/mL)	5.61 ± 0.17 a	5.25 ± 0.03 b	1.29 ± 0.08 c	ND
Y&M (lg CFU/mL)	ND	ND	ND	ND
pH	5.96 ± 0.10 a	5.94 ± 0.09 a	5.63 ± 0.03 b	4.87 ± 0.08 c
TSS (oBrix)	15.2 ± 0.3 a	14.7 ± 0.3 ab	14.4 ± 0.3 b	14.6 ± 0.4 ab
Juice yield (%)	48.9 ± 0.1	60.1 ± 1.4	60.1 ± 1.4	60.1 ± 1.4

All data were the means ± SD, *n* = 3. Different letters in the same row indicate a significant difference (*p* < 0.05). HPP: high pressure processing; TP: thermal processing; TAB: total aerobic bacteria; Y&M: yeast and molds; TSS: total soluble solids; ND: not detected.

**Table 3:** Effects of different treatments on color difference, BD and clarity of persimmon juice.

Treatments	Control	Pre-HPP (300 MPa/8 Min)	Pre-HPP + HPP (300 MPa/8 min + 550 MPa/5 Min)	Pre-HPP + TP (300 MPa/8 min + 95 °C/5 Min)
L*	35.90 ± 0.29 b	35.88 ± 0.24 b	37.39 ± 1.91 b	46.91 ± 4.39 a
a*	1.10 ± 0.04 a	0.92 ± 0.48 a	0.83 ± 0.16 a	2.18 ± 1.66 a
b*	4.66 ± 0.25 b	4.37 ± 0.32 b	2.45 ± 1.16 b	15.35 ± 5.87 a
Δ*E*	0	0.35	2.68	15.38
BD	0.585 ± 0.011 a	0.203 ± 0.028 b	0.189 ± 0.022 b	0.123 ± 0.010 c
Clarity (%)	18.2 ± 0.3 b	25.7 ± 3.3 a	18.6 ± 0.5 b	0.20 ± 0.1 c

All data were the means ± SD, *n* = 3. Different letters in the same row indicate a significant difference (*p* < 0.05). HPP: high pressure processing; TP: thermal processing; BD: browning degree.

## Data Availability

This study provided the data in the graphics and tables.
